# Factors associated with COVID-19 vaccine uptake among French population aged 65 years and older: results from a national online survey

**DOI:** 10.1186/s12877-022-03338-3

**Published:** 2022-08-02

**Authors:** Yu-Jin Jung, Amandine Gagneux-Brunon, Marion Bonneton, Elisabeth Botelho-Nevers, Pierre Verger, Jeremy K. Ward, Odile Launay

**Affiliations:** 1 Université de Paris, Inserm, Centre d’Investigation Clinique (CIC) 1417, Assistance Publique – Hôpitaux de Paris, CIC Cochin-Pasteur, Hôpital Cochin, 27 Rue du Faubourg Saint-Jacques, Paris, France; 2 Centre International de Recherche en Infectiologie, Team GIMAP, Université de Lyon, Université Jean Monnet, Université Claude Bernard Lyon 1, Inserm, U111, Centre National de Recherche Scientifique (CNRS), Unité mixte de recherche (UMR) 530, Lyon, France; 3 Inserm, CIC 1408 Vaccinologie, CHU de Saint-Etienne, Saint-Etienne, France; 4 Chaire PREVACCI, Université Jean Monnet, Saint-Etienne, France; 5 Inserm, F-CRIN, I-REIVAC/COVIREIVAC, Paris, France; 6 Observatoire Régional de la Santé (ORS) – Provence-Alpes-Côte d’Azur, Marseille, France; 7 VITROME (Aix-Marseille Université, Institut de Recherche pour le Développement, Assistance Publique – Hôpitaux de Marseille, Service de Santé des Armées), Marseille, France; 8 CERMES3 (Inserm, CNRS, Ecole des Hautes Etudes en Sciences Sociales, Université de Paris, Villejuif, France

**Keywords:** Vaccination intention, COVID-19, Pandemic

## Abstract

**Background:**

In France, the increase in COVID-19 vaccine uptake among older adults slowed down between May and June 2021. Using the data from a national survey, we aimed to assess COVID-19 vaccine uptake among French residents aged 65 years and older, particularly at risk of severe form of the infection, and identify factors associated with non-vaccination.

**Methods:**

A cross-sectional online survey collected the immunization status/intention to get the COVID-19 vaccine, reasons for vaccination/non-vaccination and factors potentially associated with vaccine uptake between May 10 and 23, 2021 among a large sample of French residents. Characteristics of participants were compared according to immunization status. Factors potentially associated with non-vaccination were computed into a multivariate logistic regression.

**Results:**

Among the 1941 survey participants, 1612 (83%) reported having received at least one dose of COVID-19 vaccine. Among the 329 unvaccinated, 197 (60%) declared having the intention to get vaccinated. Younger age (adjusted odds ratio (aOR) = 1.50; 95% confidence interval (CI), 1.05–2.14), thinking previously having COVID-19 (aOR = 4.01; 95% CI, 2.17–7.40), having suffered economic impact due to the pandemic (aOR = 2.63; 95% CI, 1.71–4.04), reporting an “unsafe” opinion about COVID-19 vaccine safety (aOR = 6.79; 95% CI, 4.50–10.26), reporting an “unsupportive” opinion about vaccination in general (aOR = 4.24; 95% CI, 2.77–6.49) were independent risk factors for non-vaccination. On the other hand, trust in COVID-19 vaccine information delivered by the doctor (aOR = 0.28; 95% CI, 0.16–0.48) and trust in the government’s actions (aOR = 0.50; 95% CI, 0.34–0.74) were independent protective factors for non-vaccination. Political affiliation also remained significantly associated with vaccine uptake.

**Conclusions:**

Despite high overall COVID-19 vaccine uptake among the study participants, differences in vaccine uptake according to the level of concerns regarding COVID-19 vaccine safety, socioeconomic profile and trust in the government were observed. Our results reinforce the importance of “reaching out” vaccination strategy that specifically targets the most vulnerable fringe of older adult population.

**Supplementary Information:**

The online version contains supplementary material available at 10.1186/s12877-022-03338-3.

## Background

In France, COronaVIrus Disease-19 (COVID-19) vaccine rollout began on December 27, 2020 and primarily aimed to contain COVID-19 morbi-mortality among the population most at risk of developing a severe infection (older adults and persons with comorbidities) and those most exposed such as health care workers [1]. Vaccine rollout first took place in nursing homes and hospitals then eligibility criteria broadened to include, since January 18, 2021, all persons aged 75 years and older and from early March, persons over 65 willing to get vaccinated. Mass vaccination centers started to set up in mid-January, mostly in urban and suburban areas, to meet the demand for vaccination. Four different vaccines were successively used after authorization by the European Medicines Agency (EMA): in chronological order, Comirnaty®/Pfizer-BioNtech, Spikevax®/Moderna, Vaxzevria®/AstraZeneca and Janssen®. At the time the data were gathered for the present study (end of May 2021), 78% of persons over 65 had received at least one dose of vaccine and 49% had received all required doses (79 and 64% for those over 75) [2]. Although vaccine uptake seems fairly high among this population, given the efforts put to making vaccination available to priority groups since January 2021, one could expect to observe even higher vaccine uptake at that point. Even to date, in early February 2022, five months through the booster campaign, there is still room for improvement in older adults’ vaccine uptake in France (94% of persons aged 65 and older completely vaccinated), while higher levels of vaccine uptake are observed in a number of neighboring European countries such as Spain and Portugal [3].

As vaccine hesitancy was declared one of the major threats to global health by the World Health Organization (WHO) [4], in the context of a pandemic where insufficient vaccine uptake could compromise efforts put worldwide to control the viral circulation and morbi-mortality, multiple stakeholders from around the globe started to assess the public’s willingness to get a future COVID-19 vaccine early before vaccine research delivered its first results. Willingness to get vaccinated was positively associated with older age, higher income and education levels, trust in the government, favorable opinion on vaccination and higher level of perceived risk and severity of COVID-19 [5–9]. As older age was widely described to be one of the most important risk factors for severe COVID-19, intention to get the COVID-19 vaccine is expected to be high among older adult population.

Older adults tend to be underrepresented in surveys about COVID-19 vaccination while they are priority targets for COVID-19 vaccination. In addition, identifying factors associated with non-vaccination is also helpful for the rollout of booster doses. Hence, the objective of our study was to asses COVID-19 vaccine uptake among adults aged 65 and older and to identify factors associated with non-vaccination.

## Methods

### Data source

A cross-sectional online survey was carried out between May 10 and 23, 2021 among a sample from a preexisting online research panel of 750,000 French residents (Bilendi SA). A quota sampling method was applied to obtain a sample of 1514 participants aged 18 years and older selected to match the general population structure in terms of gender, age, region, size of residence area and occupation according to the last available national census [10] and 1544 additional French residents 65 years of age and older selected from the same panel, representative of the general “senior” population in terms of gender and age. For the present study, we pooled the data collected from all participants aged 65 years and over (397 participants from the general population sample and 1544 from the senior population sample). Data were weighted according to gender, age, region and size of residence area. The study protocol was approved by the ethics committee of the Institut Hospitalo-Universitaire – Méditerranée Infection (#2021–001).

### Data collection and processing

The survey aimed to collect the immunization status of participants against COVID-19 (“Already got the vaccine”, “Will certainly get the vaccine”, “Will probably get the vaccine”, “Will probably not get the vaccine” and “Will certainly not get the vaccine”). The outcome of interest was defined as being unvaccinated (immunization status different from “Already got the vaccine”). Participants were asked to choose three reasons, in order of priority, for vaccination/non-vaccination from a list of options based on the results of previous surveys (reasons for vaccination: “To protect myself”, “To protect my family and friends”, “To protect the most vulnerable”, “To resume a “normal” life as soon as possible”, “To get the country out of an economic crisis”; reasons for non-vaccination: “I am against vaccination in general”, “A vaccine developed in a hurry is too dangerous”, “It is useless after all, COVID-19 is not very dangerous”, “It is too difficult to get a vaccination appointment”). Collected factors potentially associated with vaccine uptake were as follows: first, the participants were asked about their personal experience with the COVID-19 pandemic (history of infection, impact of pandemic on a personal level), habits and their level of concern about getting the COVID-19 (or getting it again) on a scale of 0 (not concerned at all) to 10 (extremely concerned). Second, opinions and perceptions about the available COVID-19 vaccines (perceptions about COVID-19 vaccines’ efficacy/safety, level of trust in different sources of information) and vaccination in general were collected. Third, participants were asked to report their political opinion (level of trust in current government, political party they felt the closest to among a list of 17 parties plus “Other” and “Neither” options). Finally, participants were shown the list of comorbidities associated with an increased risk of severe COVID-19 (obesity, diabetes, chronic heart failure, chronic renal insufficiency, chronic obstructive pulmonary disease and other chronic respiratory diseases, complicated hypertension, active cancer, solid organ or stem cell transplantation) and confirmed if they presented at least one comorbidity from the list.

The level of concern about getting infected by SARS-CoV-2 was recoded as a quartile-split categorical variable (0–2 = “Unconcerned or very little concerned”; 3–5 = “Moderately concerned”; 6–7 = “Quite concerned”; 8–10 = “Very concerned”) with an “Unsure” option. Health and economic impacts due to the pandemic and the level of trust in different sources of information were collected using four-level Likert scales, then were converted into binary variables (“Yes” and “No”) for the present study. COVID-19 vaccine efficacy/safety opinions were initially collected using five-level Likert scales including a neutral option (“Unsure”) for each available COVID-19 vaccine in France (What do you think about the efficacy/safety of (Comirnaty®, Vaxzevria®, Spikevax®, Janssen®)?). A score was assigned to each response (“Very effective/safe” = 2; “Somewhat effective/safe” = 1; “Unsure” = 0; “Somewhat uneffective/unsafe” = −1; “Uneffective/unsafe” = −2) and a global efficacy/safety score was calculated by summing up the responses for the four vaccines. The general opinion on COVID-19 vaccine was considered to be “Effective/safe” when the sum was >0; “Unsure” if 0 and “Uneffective/unsafe” if <0. Responses concerning the political affiliation were grouped into an eight-level variable: “Far-Left”, “Left”, “Green”, “Center” (including “La République En Marche!” , current governing party), “Right”, “Far-Right”, “Other” and “Neither”.

The survey questionnaire is available (French translated into English) as supplementary material.

### Statistical analysis

Characteristics of participants according to COVID-19 immunization status were compared using χ^2^ test and univariate logistic regression. Factors potentially associated with non-vaccination were selected based on prior knowledge [9, 11, 12]. Those with a *p*-value<0.10 were computed into a multivariate logistic regression with all collected sociodemographic characteristics. When two or more variables were correlated or expressed a similar idea, the variable with the smaller *p*-value or considered by the authors to be more clinically relevant, was retained in the model. For the purpose of multivariate logistic regression, categories with low numbers were grouped and categorical variables were recoded into binary variables (“Yes” or “No”), with a neutral option (“Unsure”) when relevant. The significance threshold was fixed at 0.05. All statistical analyses were performed on weighted responses using Stata Statistical Software (Release 14. College Station, TX: StataCorp LP).

## Results

### Survey participants’ characteristics, immunization status/intention to get vaccinated

The overall characteristics of survey participants are shown in Table 1. Women (57%) and persons aged 65–74 years (54%) were slightly predominant as observed in the general population. The majority of participants lived in areas where the population did not exceed 20,000 inhabitants and had had white collar jobs. Five percent of the participants (100) thought they previously had COVID-19. For 77 of them, the diagnosis was confirmed by a doctor or a test. Five hundred and ninety-nine respondents (31%) declared having suffered health impact due to the pandemic and 208 (11%) declared having suffered economic impact.

**Table 1:** Individual characteristics of survey respondents (*n* = 1941) overall and according to immunization status

Individual characteristics	Overall (1941)^a^		Vaccinated (1612)^a^		Unvaccinated (329)^a^		p^b^
	n	%	n	%	n	%	
Gender							
Female	1105	57	890	55	215	65	0.001
Male	836	43	722	45	114	35	
Age category							
65–74	1049	54	839	52	210	64	<0.001
75+	892	46	773	48	119	36	
Region							
Auvergne-Rhône-Alpes	237	12	208	13	29	9	0.018
Bourgogne-Franche-Comté	96	5	86	5	10	3	
Bretagne	112	6	89	6	23	7	
Centre-Val de Loire	85	4	66	4	19	6	
Grand Est	167	9	150	9	16	5	
Hauts-de-France	162	8	139	9	23	7	
Ile-de-France	271	14	225	14	45	14	
Normandie	107	5	93	6	13	4	
Nouvelle Aquitaine	214	11	173	11	41	12	
Occitanie	200	10	158	10	42	13	
Pays de la Loire	118	6	88	5	30	9	
Provence-Alpes-Côte d’Azur	173	9	136	8	37	11	
Population of residence area							
< 2000 inhabitants	549	28	431	27	118	36	0.005
2000–20,000 inhabitants	795	41	681	42	114	35	
20,000–100,000 inhabitants	376	19	321	20	55	17	
> 100,000 inhabitants	221	11	179	11	42	13	
Former occupation							
Farmer	4	<1	4	<1	0	–	0.004
Craftman, merchant, company director	104	5	78	5	26	8	
Executive or higher intellectual profession	843	43	731	45	112	34	
Intermediate profession	534	28	442	27	92	28	
Employee	358	18	283	18	75	23	
Worker	83	4	65	4	18	6	
Other non-working people	16	<1	10	1	6	2	
At least one comorbidity associated with increased risk of severe COVID-19							
Yes	849	44	723	45	125	38	0.072
No	1030	53	841	52	189	58	
Unsure	63	3	48	3	15	4	
**Perception, knowledge and personal experience with COVID-19**							
Think previously had COVID-19 (Yes)	100	5	62	4	38	11	<0.0001
Concern about catching the COVID-19							
Unconcerned or very little concerned	447	23	387	24	60	18	0.037
Moderately concerned	574	30	469	29	105	32	
Quite concerned	429	22	365	23	64	20	
Very concerned	377	19	306	19	71	22	
Unsure	116	6	87	5	29	9	
Suffered health impact due to the pandemic (Yes)	599	31	475	29	125	38	0.005
Suffered economic impact due to the pandemic (Yes)	208	11	139	9	69	21	<0.0001
Following-up news on COVID-19							
Daily	1262	65	1094	68	168	51	<0.0001
Several times a week	328	17	267	17	60	18	
Once or twice a week	169	9	126	8	44	13	
Less often	145	7	105	7	39	12	
Never	38	2	21	1	17	5	
**Attitudes and perceptions about COVID-19 vaccines**							
Trust in information on vaccines delivered by:							
Doctor (Yes)	1826	94	1561	97	266	81	<0.0001
Pharmacist (Yes)	1685	87	1464	91	221	67	<0.0001
Ministry of health (Yes)	1138	59	1040	65	98	30	<0.0001
Government (Yes)	940	48	874	54	66	20	<0.0001
Pharmaceutical industry (Yes)	858	44	789	49	69	21	<0.0001
Experts visible in the media (Yes)	1200	62	1078	67	122	37	<0.0001
Family and friends (Yes)	1342	69	1142	71	200	61	0.0011
The internet (Yes)	302	16	252	16	50	15	0.859
Traditional media (Yes)	805	42	736	46	70	21	<0.0001
Public health agencies (Yes)	1213	63	1101	68	112	34	<0.0001
General opinion about COVID-19 vaccine efficacy							
Effective	1564	81	1406	87	158	48	<0.0001
Uneffective	99	5	30	2	69	21	
Unsure	278	14	176	11	102	31	
General opinion about COVID-19 vaccine safety							
Safe	1398	72	1284	80	114	35	<0.0001
Unsafe	245	13	112	7	133	40	
Unsure	299	15	216	13	82	25	
Attitude towards vaccination in general							
Very supportive	843	43	784	49	60	18	<0.0001
Somewhat supportive	826	43	687	43	139	42	
Somewhat unsupportive	157	8	93	6	64	19	
Very unsupportive	71	4	25	2	46	14	
Unsure	44	2	24	1	20	6	
**Political beliefs**							
Trust in government’s actions to control the epidemic (Yes)	1012	52	925	57	87	27	<0.0001
Political party affiliation							
Far-Left	107	5	83	5	23	7	<0.0001
Left	243	13	213	13	30	9	
Green	98	5	76	5	22	7	
Center	456	24	405	25	50	15	
Right	337	17	301	19	36	11	
Far-Right	212	11	145	9	67	20	
Other	25	1	19	1	6	2	
Neither	464	24	370	23	94	29	

^a^As analyses were carried out on weighted data and underwent rounding, the sums, as well as percentages, for each lign and column can slightly differ from the expected totals

^b^*P*-values resulting from comparison of vaccinated and unvaccinated participants

Among the 1941 participants, 1612 (83%) reported having received at least one dose of COVID-19 vaccine (80% among participants aged 65–74 and 87% for participants aged 75 and older). The majority of the vaccinated participants (75%) received Comirnaty®, 16% Vaxzevria®, 8% Spikevax® and a very few got the Janssen® vaccine. Four participants were not able to recall which vaccine they had received. Among the 329 (17%) unvaccinated participants, 100 (30%) declared they would “Certainly get the vaccine”, and 97 (30%) would “Probably get the vaccine”. Sixty-six (20%) responded that they would “Probably not get the vaccine” and the same number that they would “Certainly not get the vaccine”.

General opinions on COVID-19 vaccine efficacy and safety were rather favorable; efficacy obtained slightly more favorable opinions than safety (81% vs 72%). Unfavorable opinions were more frequently reported for safety compared to efficacy (13% vs 5%). Besides, a notable proportion of participants were unsure about the efficacy/safety of COVID-19 vaccines (14 and 15% respectively). Attitude towards vaccination in general was rather supportive (86%) and the proportion of unsure opinions was much lower (2%). Half of participants reported trusting the government’s actions to control the epidemic.

### Factors associated with non-vaccination and reasons for vaccination/non-vaccination

Factors associated with non-vaccination are shown in Tables 1 and 2. Participants aged 65–74 years were more frequently unvaccinated compared to those aged 75 and older. Participants living in small localities (<2000 inhabitants) were more frequently unvaccinated compared to participants living in areas counting 2000 to 20,000 inhabitants. Formerly self-employed, former employees and workers were more frequently unvaccinated compared to former executives. Proportion of unvaccinated was higher among participants who reported having suffered health and/or economic impact from the pandemic. In terms of political affiliation, the proportion of unvaccinated was higher among participants reporting feeling closest to Far-Left/Far-Right, Green, “Other” and “Neither” parties.

**Table 2:** Univariate and multivariate analyses of factors associated with non-vaccination

	Unvaccinated %	Crude OR for non-vaccination		*p*	Adjusted OR for non-vaccination		*p*
Age category							
65–74	20	1.63	1.22–2.16	<0.001	1.50	1.05–2.14	0.025
75+ (reference)	13	1			1		
Female (vs male)	20	1.53	1.18–1.98	0.001	0.94	0.67–1.32	0.723
Region							
Auvergne-Rhône-Alpes	12	0.69	0.43–1.13	0.018	0.57	0.31–1.05	0.078
Bourgogne-Franche-Comté	11	0.58	0.26–1.29		0.61	0.21–1.76	
Bretagne	21	1.29	0.72–2.34		0.93	0.44–1.95	
Centre-Val de Loire	22	1.43	0.78–2.62		1.43	0.67–3.03	
Grand Est	10	0.54	0.28–1.07		0.47	0.22–1.00	
Hauts-de-France	14	0.81	0.44–1.47		0.69	0.33–1.43	
Ile-de-France (reference)	17	1			1		
Normandie	13	0.72	0.31–1.68		0.91	0.37–2.29	
Nouvelle Aquitaine	19	1.18	0.73–1.89		1.20	0.65–2.20	
Occitanie	21	1.32	0.83–2.09		1.43	0.80–2.57	
Pays de la Loire	26	1.70	0.99–2.90		1.32	0.65–2.68	
Provence-Alpes-Côte d’Azur	21	1.35	0.83–2.18		1.20	0.65–2.22	
Population of residence area							
< 2000 inhabitants	21	1.63	1.17–2.27	0.005	1.78	1.18–2.68	0.053
2000–20,000 inhabitants (reference)	14	1			1		
20,000–100,000 inhabitants	15	1.03	0.71–1.48		1.20	0.76–1.88	
> 100,000 inhabitants	19	1.42	1.01–1.99		1.31	0.85–2.02	
Former occupation							
Farmer, craftman, merchant, company director	24	2.08	1.20–3.59	0.004	1.99	0.99–3.98	0.086
Executive or higher intellectual profession (reference)	13	1			1	1	
Intermediate profession	17	1.36	0.98–1.87		1.09	0.73–1.61	
Employee	21	1.73	1.22–2.46		1.37	0.89–2.11	
Worker	22	1.84	1.02–3.31		0.93	0.41–2.13	
Other non-working people	36	3.67	1.07–12.6		8.43	1.27–55.84	
At least one comorbidity associated with risk of severe COVID-19							
Yes	15	0.77	0.59–1.01	0.072	0.83	0.59–1.17	0.343
No (reference)	18	1			1		
Unsure	23	1.36	0.71–2.60		0.61	0.25–1.45	
Think previously had COVID-19 (unconfirmed) (yes vs no)	38	3.21	2.05–5.02	<0.0001	4.01	2.17–7.40	<0.001
Concern about catching the COVID-19							
Concerned (reference)	17	1		0.098	1		0.862
Unconcerned	16	0.96	0.73–1.26		1.10	0.78–1.55	
Unsure	25	1.65	1.00–2.71		1.04	0.52–2.06	
Suffered economic impact due to the pandemic (yes vs no)	33	2.82	1.99–3.98	<0.0001	2.63	1.71–4.04	<0.001
Following-up news on COVID-19 at least once a week (yes vs no)	16	0.41	0.28–0.59	<0.0001	1.21	0.71–2.08	0.480
General opinion about COVID-19 vaccine safety							
Safe (reference)	9	1		<0.0001	1		<0.001
Unsafe	38	6.54	4.80–8.91		6.79	4.50–10.26	
Unsure	23	3.25	2.26–4.66		2.78	1.88–4.09	
Attitude towards vaccination in general							
Supportive (reference)	12	1		<0.0001	1		<0.001
Unsupportive	48	6.94	5.00–9.64		4.24	2.77–6.49	
Unsure	46	6.23	3.22–12.07		5.12	1.98–13.23	
Trust in information on vaccines delivered by doctor (yes vs no)	15	0.14	0.09–0.21	<0.0001	0.28	0.16–0.48	<0.001
Trust in government’s actions to control the epidemic (yes vs no)	9	0.27	0.20–0.36	<0.0001	0.50	0.34–0.74	0.001
Political party affiliation							
Far-Left	22	2.21	1.22–4.03	<0.0001	1.12	0.52–2.38	0.043
Left	13	1.15	0.68–1.95		0.70	0.37–1.32	
Green	23	2.34	1.25–4.39		0.89	0.41–1.93	
Center (reference)	11	1			1		
Right	11	0.96	0.58–1.60		0.40	0.21–0.76	
Far-Right	32	3.75	2.37–5.94		1.02	0.55–1.88	
Other	25	2.72	0.92–8.03		0.79	0.16–3.81	
Neither	20	2.06	1.35–3.14		0.66	0.38–1.14	

In multivariate analysis, younger age remained a risk factor for non-vaccination (age 65–74 vs 75 and older: adjusted odds ratio (aOR) = 1.50; 95% confidence interval (CI), 1.05–2.14). The size of residence area and former occupation were no longer significantly associated with non-vaccination, although participants living in small localities tended to be less vaccinated than those living in areas counting 2000 to 20,000 inhabitants (aOR = 1.78; 95% CI, 1.18–2.68). Other independent risk factors for non-vaccination were: thinking previously having COVID-19 (aOR = 4.01; 95% CI, 2.17–7.40), having suffered economic impact due to the pandemic (aOR = 2.63; 95% CI, 1.71–4.04), reporting an unsafe/unsure opinion about COVID-19 vaccine safety (unsafe aOR = 6.79; 95% CI, 4.50–10.26; unsure aOR = 2.78; 95% CI, 1.88–4.09), reporting an unsupportive/unsure opinion about vaccination in general (unsupportive aOR = 4.24; 95% CI, 2.77–6.49; unsure aOR = 5.12; 95% CI, 1.98–13.23). On the other hand, trust in COVID-19 vaccine information delivered by the doctor (aOR = 0.28; 95% CI, 0.16–0.48) and trust in the government’s actions (aOR = 0.50; 95% CI, 0.34–0.74) were found to be independent protective factors for non-vaccination. Political affiliation remained significantly associated with non-vaccination.

Reasons for vaccination and non-vaccination are shown in Tables 3 and 4. Self-protection was reported as the most frequent motivation (54%) to get vaccinated. Safety concerns about vaccines developed “in a hurry” were found to be the most frequent reason for non-vaccination (67%).

**Table 3:** First reason for vaccination among vaccinated participants and those willing to get the vaccine

	Vaccinated participants (1612)		Participants declaring they “Will certainly get the vaccine” (100)		Participants declaring they “Will probably get the vaccine” (97)	
	n	%	n	%	n	%
To protect myself	869	54	43	43	40	42
To protect my family and friends	282	18	23	22	22	23
To protect the most vulnerable	98	6	7	7	4	4
To resume a “normal” life as soon as possible	282	18	19	19	20	20
To get the country out of an economic crisis	67	4	9	9	3	3
Other	15	<1	1	<1	8	8

**Table 4:** First reason for non-vaccination among participants not willing to get the vaccine

	Participants not willing to get the vaccine (132)		Participants declaring they “Will certainly not get the vaccine” (66)		Participants declaring they “Will probably not get the vaccine” (66)	
	n	%	n	%	n	%
I am against vaccination in general	17	13	15	22	2	4
A vaccine developed in a hurry is too dangerous	88	67	42	64	46	70
It is useless after all, COVID-19 is not very dangerous	4	3	4	6	1	1
It is too difficult to get a vaccination appointment	2	1	1	2	1	1
Other	21	2	4	7	16	25

## Discussion

The present study, conducted 2 months after COVID-19 vaccination was made available to all persons aged 65 years and older (end of May 2021) in France, shows a reported vaccine uptake for at least one dose of 83% among the participants (80% among participants aged 65–74 and 87% for participants aged 75 and older). Intention to get vaccinated among unvaccinated participants was high (60%) as well as supportive attitude towards vaccination in general (86%). Participants living in small localities (<2000 inhabitants) were more likely to be unvaccinated compared to all other residence areas, suggesting that geographic accessibility to COVID-19 vaccination probably remained an issue in May 2021. The most frequently reported reason for non-vaccination was linked to safety concerns towards vaccines developed in a hurry as reported by unvaccinated (or unwilling) groups from previous French surveys, including younger adults, and multiple other surveys from different parts of the world [8, 13–17].

Data specifically focusing on the older adult population group, particularly at risk of severe COVID-19 remain scarce. The study by Callow et al., carried out on a sample of adults aged 60 and older in the United States before COVID-19 vaccine rollout took place, confirmed the COVID-19 vaccine uptake predictors among this population were comparable to those observed in the general adult population [18]. In fact, in the present study, several factors already known to predict vaccine uptake such as age, opinion on COVID-19 vaccines and vaccination in general and the level of confidence in healthcare and politics were confirmed to be independently associated with COVID-19 vaccine uptake. Politicization of COVID-19 vaccination was previously described by Ward et al. in France [13, 19]; our results showed that COVID-19 vaccine uptake tended to be higher among participants who felt closest to “mainstream” governing parties compared to the partisans of Far-Left/Far-Right parties and those with no declared political affiliation. Reporting having suffered economic impact due to the pandemic was found to be an independent risk factor for non-vaccination. Participants declaring economic impact tended to be younger, more frequently women, living in small localities and formerly self-employed (education and income levels were not available for the present study). This apparently paradoxical observation could be compared to what was reported in a previous study assessing French general population’s support level of the first lockdown [20]. In fact, Peretti-Watel et al. showed that those who suffered the most impact from the control measures implemented by the government to mitigate the epidemic wave, who were found to be more frequently displaying low-income and low-education profiles, were more likely to display critical views towards said measures. High socioeconomic status is usually associated with a higher level of trust in public health authorities, as illustrated by public reactions during the 2009 H_1_N_1_ pandemic [21]. While one might have expected that socioeconomic factors would be outweighted by the high perceived risk of severity of COVID-19 among older adults in making the decision to get vaccinated, our results show that vaccine uptake remains affected by socioeconomic vulnerability. A recent systematic review collecting data from survey participants aged 60 and older reported that unwillingness to get vaccinated against the COVID-19 was indeed higher among low-income and low-education profiles [22].

On the other hand, the presence of comorbidities and the level of concern about catching (or recatching) the COVID-19 were not found to be independently associated with vaccine uptake, while awareness, perceived risk or susceptibility and severity of the disease are often found to be determinants of vaccine uptake in general [11, 23, 24] and intention to get the COVID-19 vaccine [5, 8, 25]. Awareness and perceived risk of severity of COVID-19 may be intrinsically high among the participants, independently from underlying medical conditions because of their older age.

Besides, our results showed a very high overall level of trust in COVID-19 vaccine information delivered by local healthcare workers (94% trust in general practitioner and 87% in pharmacist vs 48% in government) even though it was lower among unvaccinated participants. Local healthcare workers, who contribute to the COVID-19 vaccine rollout, either in their practice or on mass vaccination sites, play a key role in convincing and accompanying their patients in the decision to get the vaccine, especially among the older adult population [18]. In addition, it is important to highlight the fact that the Internet was rarely reported to be a trustful source of information on COVID-19 vaccines (16% overall) by the participants, regardless of immunization status.

France was cited as one of the most reluctant countries regarding vaccination [26]. Nonetheless, as shown in precedent surveys [13, 14], vaccine reluctance gradually decreased as COVID-19 vaccine rollout began. France managed to achieve a relatively high COVID-19 vaccine uptake among the adult population with the support and implication of healthcare workers of different backgrounds, as opposed to what was observed during the 2009 H_1_N_1_ pandemic, until mid-June 2021. As COVID-19 vaccine uptake was plateauing, the French Government made the COVID-19 certificate (either complete COVID-19 vaccination or negative test within 72 hours or proof of recovery from COVID-19 episode within 6 months) mandatory to travel and access hospitals, cultural venues, restaurants and bars starting from July 21, 2021. To date, in early February 2022, 94% of persons aged 65 years and older received at least one dose of vaccine and 93% are completely vaccinated [2]. COVID-19 mandatory certificate played an important role in pushing the complacent adults to make the appointment for vaccination. Yet, its impact among older adults, particularly those living in rural areas was probably much less because of their social activity patterns that can differ substantially from those living in the urban/suburban areas. In response to the threat of Omicron variant, presumably more transmissible and prone to immune escape [27, 28], the French Government has passed a new law, early January 2022, to turn the COVID-19 certificate into a COVID-19 vaccine certificate (either complete COVID-19 vaccination or proof of recovery from COVID-19 episode within 3 months), which now includes one booster dose, considered essential to maintain the efficacy of COVID-19 vaccines in preventing severe forms of the infection [29]. According to the past experience of COVID-19 certificate, the deployment of the COVID-19 vaccine certificate might not lead to a spectacular increase of vaccine uptake among the aforementioned older adults [30].

The need for reaching the final segment of the unvaccinated population, especially those most at risk of severe COVID-19, among whom older adults, remains a priority. Our results show that despite a high level of vaccine uptake and favorable opinions on COVID-19 vaccines among the participants, a part of them still expressed doubts and concerns about the safety of said vaccines. Although one could have expected that the health benefits of COVID-19 vaccines would prevail among the older adults, vaccine uptake remained affected by socioeconomic conditions and political consideration, as observed in the general adult population. Furthermore, among older adults aged 65 and older, disparities are observed in vaccine uptake: persons aged 65–69 remain less vaccinated (91% completely vaccinated) than persons aged 70–79 (99%) then vaccine uptake decreases for persons aged 80 and older (87%). Persons aged 80 and older are now the least vaccinated age group among the adult population in France (for instance, complete vaccine uptake is 94, 93 and 89% for age groups 18–24, 25–29 and 30–39 respectively and 80% for children aged 12–17) [2]. Delta, then Omicron surges complexified the COVID-19 vaccination strategy as they required accelerating the rollout of booster doses while also laboriously catching up on primary vaccination.

Vaccine uptake is high in nursing home residents (93%) and the youngest of older adults living in urban/suburban areas who are familiarized with new technologies (online vaccination appointments, QR code for COVID-19 certificate) that were extensively used for mass COVID-19 vaccine rollout in France. In fact, COVID-19 vaccine rollout did not benefit all older adult population, especially those living at home in rural areas. Along with persons presenting socioeconomic vulnerability, as observed in the present study, the oldest unvaccinated group may benefit from “reaching out” measures [31], particularly those involving the local healthcare workers, who are trusted sources of information regarding COVID-19 vaccination. The availability of Comirnaty®, which was delivered only on mass vaccination sites, at the general practitioners’ practices, dispensing ready-to-use single doses of vaccine to a wide range of healthcare workers by local pharmacies, mobile vaccination units and at-home vaccinations are some examples of reaching out measures.

To our knowledge, the present study is the first in France and one of the few reported in the literature to specifically address the issue of COVID-19 vaccine uptake among older adults, with a relatively large sample size. The study sample consisted of participants selected from a general population sample and an additional older adult population sample representative of French residents aged 65 years and older in terms of gender and age. However, the survey was carried out online, which favored the recruitment of participants with a high level of digital literacy. Persons aged 80 and older are slightly underrepresented and the survey was more frequently completed by former executives or persons having worked higher intellectual professions (vs former employees and workers). As a consequence, our results tend to overestimate vaccine uptake and favorable opinions towards vaccination.

## Conclusions

Factors associated with non-vaccination in older adults are not very different from those identified in the general adult population. Although vaccine uptake and intention to get the COVID-19 vaccine were high, vaccine uptake among the study participants differed according to their opinion on COVID-19 vaccine safety, socioeconomic profile and trust in the government. Our results reinforce the importance of “reaching out” vaccination strategy, mostly relying on local healthcare workers who are in close contact with vulnerable older adults.

## Supplementary Information

**Additional file 1.**

## Data Availability

Dataset can be shared upon reasonable request sent to the corresponding author. The survey questionnaire is available (French translated into English) as supplementary material. The author(s) read and approved the final manuscript.
