# Supplementary material for: Factors associated with COVID-19 vaccine uptake among French population aged 65 years and older: results from a national online survey
Source: BMC Geriatr. 2022 Aug 2;22:637. doi: 10.1186/s12877-022-03338-3 (PMC9344255; doi:10.1186/s12877-022-03338-3)
Supplement: Supplementary file 1 — Additional file 1. [file 12877_2022_3338_MOESM1_ESM.pdf]

COVIREIVAC survey – 65 years and older

Age category
65 - 74 years old
75 years and older

You are:
Male
Female

In which region do you live?
Auvergne-Rhône-Alpes
Bourgogne-Franche-Comté
Bretagne
Centre-Val de Loire
Grand Est
Hauts-de-France
Ile-de-France
Normandie
Nouvelle Aquitaine
Occitanie
Pays de la Loire
Provence-Alpes-Côte d'Azur

You live in a locality with a population
Less than 2 000 inhabitants (rural)
2 000 to 20 000 inhabitants (for example: Marmande, Verdun, Douarnenez …)
20 000 à 100 000 habitants (for example: Antony, Chambéry, Poitiers, Valence, Calais …)
Of more than 100 000 habitants (for example: Grenoble, Toulouse, Lille, Strasbourg, Lyon, Marseille, Montreuil, Paris …)

Current or former occupation
Farmer
Craftman, merchant, company director
Executive or higher intellectual profession
Intermediate profession
Employee
Worker
Other non-working people

Do you think you have ever had COVID-19?
Yes
No

Has this been confirmed by a test or by a doctor?
Yes
No

On a scale of 0 to 10, how concerned are you about the possibility of [catching /recatching] COVID-19 by the end of the epidemic?
0
1
2
3
4
5
6
7
8
9
10
You don't know

Has the health crisis had a negative impact on your health (delays in care, anxiety, depression, sleeping problems, …)?
Yes, absolutely
Yes, somewhat
No, not really
No, not at all

Has the health crisis had a negative impact on your economic situation?
Yes, absolutely
Yes, somewhat
No, not really
No, not at all

How often do you currently follow news on COVID-19?
Daily
Several times a week
Once or twice a week
Less often
Never

Do you plan to get the coronavirus vaccine at some point?

Yes, certainly
Yes, probably
No, probably not
No, certainly not
You are already vaccinated (at least one dose received)

Which vaccine have you received?

Astrazeneca's vaccine
Pfizer's vaccine
Moderna's vaccine
Janssen's vaccine (Johnson and Johnson)
You don't know

There are many reasons why you may want to be vaccinated. Among the following reasons, what are your main motivations?

To protect myself
To protect my family and friends
To protect the most vulnerable
To resume a "normal" life as soon as possible
To get the country out of an economic crisis
Other
None of these reasons
You don't know

There are many reasons why you may want to be vaccinated. Among the following reasons, what are your main motivations?

To protect myself
To protect my family and friends
To protect the most vulnerable
To resume a "normal" life as soon as possible
To get the country out of an economic crisis
Other
None of these reasons
You don't know

There are many reasons why you may want to be vaccinated. Among the following reasons, what are your main motivations?

To protect myself
To protect my family and friends
To protect the most vulnerable
To resume a "normal" life as soon as possible
To get the country out of an economic crisis
Other
None of these reasons
You don't know

What are the reasons why you would not get vaccinated?

Because I am against vaccination in general
Because a vaccine developed in a hurry is too dangerous
Because it is useless after all, COVID-19 is not very dangerous
Because it is too difficult to get a vaccination appointment
Other

What are the reasons why you would not get vaccinated?

Because I am against vaccination in general
Because a vaccine developed in a hurry is too dangerous
Because it is useless after all, COVID-19 is not very dangerous
Because it is too difficult to get a vaccination appointment
Other

What are the reasons why you would not get vaccinated?

Because I am against vaccination in general
Because a vaccine developed in a hurry is too dangerous
Because it is useless after all, COVID-19 is not very dangerous
Because it is too difficult to get a vaccination appointment
Other

For each of the following vaccines, tell us what you think about their efficacy: Astrazeneca

Very effective
Somewhat effective
Somewhat uneffective
Uneffective
You don't know

For each of the following vaccines, tell us what you think about their efficacy: Pfizer

Very effective
Somewhat effective
Somewhat uneffective
Uneffective
You don't know

For each of the following vaccines, tell us what you think about their efficacy: Moderna

Very effective
Somewhat effective

Somewhat uneffective
Uneffective
You don’t know

For each of the following vaccines, tell us what you think about their efficacy: Janssen

Very effective
Somewhat effective
Somewhat uneffective
Uneffective
You don’t know

For each of the following vaccines, tell us what you think about their safety: Astrazeneca

Very safe
Somewhat safe
Somewhat unsafe
Unsafe
You don’t know

For each of the following vaccines, tell us what you think about their safety: Pfizer

Very safe
Somewhat safe
Somewhat unsafe
Unsafe
You don’t know

For each of the following vaccines, tell us what you think about their safety: Moderna

Very safe
Somewhat safe
Somewhat unsafe
Unsafe
You don’t know

For each of the following vaccines, tell us what you think about their safety: Janssen

Very safe
Somewhat safe
Somewhat unsafe
Unsafe
You don’t know

How much do you trust the following sources of information about COVID-19 vaccines? Your doctor

Fully trusting
Somewhat trusting
Not really trusting
Not trusting at all

How much do you trust the following sources of information about COVID-19 vaccines? Your pharmacist

Fully trusting
Somewhat trusting
Not really trusting
Not trusting at all

How much do you trust the following sources of information about COVID-19 vaccines? The ministry of health

Fully trusting
Somewhat trusting
Not really trusting
Not trusting at all

How much do you trust the following sources of information about COVID-19 vaccines? The government

Fully trusting
Somewhat trusting
Not really trusting
Not trusting at all

How much do you trust the following sources of information about COVID-19 vaccines? The pharmaceutical industry

Fully trusting
Somewhat trusting
Not really trusting
Not trusting at all

How much do you trust the following sources of information about COVID-19 vaccines? Experts visible in the media

Fully trusting
Somewhat trusting
Not really trusting
Not trusting at all

How much do you trust the following sources of information about COVID-19 vaccines? Family and friends

Fully trusting
Somewhat trusting
Not really trusting
Not trusting at all

How much do you trust the following sources of information about COVID-19 vaccines? The Internet

Fully trusting
Somewhat trusting

Not really trusting
Not trusting at all

How much do you trust the following sources of information about COVID-19 vaccines? Traditional media (newpapers, television, radio)

Fully trusting
Somewhat trusting
Not really trusting
Not trusting at all

How much do you trust the following sources of information about COVID-19 vaccines? Public health agencies (Santé publique France, Agence Nationale de Sécurité du Médicament)

Fully trusting
Somewhat trusting
Not really trusting
Not trusting at all

How much do you trust the government to effectively combat the coronavirus outbreak?

Fully trusting
Somewhat trusting
Not really trusting
Not trusting at all

What is your attitude towards vaccination in general?

Very supportive
Somewhat supportive
Somewhat unsupportive
Very unsupportive
You don't know

Which of the following political formations do you feel closest to or least distant from?

Lutte Ouvrière
Le Nouveau Parti Anticapitaliste d'Olivier Besancenot
La France Insoumise de Jean-Luc Mélenchon
Le Parti Communiste
Génération.s de Benoît Hamon
Le Parti Socialiste
Europe Ecologie Les Verts
La République en Marche
Le MoDem
Le Parti Radical
L'UDI (Union des Démocrates et Indépendants) de Jean-Christophe Lagarde
Agir, la droite constructive
Les Républicains
Debout La France de Nicolas Dupont-Aignan
Union Populaire Républicaine de François Asselineau
Les Patriotes de Florian Philippot
Le Rassemblement National
Neither
Other

Do you have one or more of the following risk factors? (obesity, diabetes, chronic heart failure, chronic renal insufficiency, chronic obstructive pulmonary disease and other chronic respiratory diseases, complicated hypertension, active cancer, solid organ or stem cell transplantation)

Yes
No
You don't know
